# The association between ADAMTS14/rs4747096 gene polymorphism and some risk factors and knee osteoarthritis

**DOI:** 10.1186/s12891-024-07943-8

**Published:** 2024-10-30

**Authors:** Ghada A. Elshaarawy, Iman I. Salama, Somaia I. Salama, Amany H. Abdelrahman, Mirhane Hassan, Eman Eissa, Sherif Ismail, Sherif E. Eldeeb, Doaa E. Ahmed, Hazem Elhariri, Rasmia Elgohary, Aida M. Abdelmohsen, Walaa A. Fouad, Hala M. Raslan

**Affiliations:** 1 Community Medicine Research Department, Medical Research and Clinical Studies Institute, National Research Centre, Dokki, P.O. 12622, Giza, Egypt; 2 Clinical & Chemical Pathology Department, Medical Research and Clinical Studies Institute,, National Research Centre, Dokki, P.O. 12622, Giza, Egypt; 3 Immunogenetics Department, Human Genetics and Genome Research Institute, National Research Centre, Dokki, P.O. 12622, Giza, Egypt; 4 Internal Medicine Department, Medical Research and Clinical Studies Institute, National Research Centre, Dokki, P.O. 12622, Giza, Egypt; 5 Rheumatology and Immunology Unit, Internal Medicine Department, Kasr Alainy School of Medicine, Cairo University, El-Maniel, P.O. 11562, Cairo, Egypt

**Keywords:** KOA, ADAMTS14 gene rs4747096, Genotypes, Alleles, And WOMAC

## Abstract

Knee osteoarthritis (KOA) is an important cause of disability in the world and it denotes a public health defiance of the upcoming years.

**Aim **To examine the connection between ADAMTS14 gene rs4747096 polymorphism and KOA and to assess risk factors associated with KOA.

**Methods **A case control study was conducted on 158 patients with KOA and 120 controls with comparable age and sex randomly recruited from National Research Centre employees. All participants were subjected to full history taking, assessment of KOA severity using WOMAC scoring system, and thorough clinical examination. Blood sample was collected for detection of ADAMTS14/rs4747096 gene polymorphism.

**Results **The frequency of ADAMTS14 gene rs4747096 genotypes among patients with KOA was 73.5% for AA, 25.7% for AG, and 0.7% for GG compared to controls 963%, 31.3%, and 5.6% respectively and the frequency of alleles among patients was 86.4% for A and 78.7% for G compared to controls (78.7% and 21.3% respectively, *P* < 0.05. The study found that the median levels of total WOMAC score and its domains were significantly higher among KOA patients than controls. The logistic regression analysis revealed that age ≥ 50 years, BMI ≥ 35, and long standing at work were predictive factors for KOA (*P* < 0.05). Regarding different genetic patterns, only the A recessive pattern of inheritance was found to be a predictive risk factor for KOA.

**Conclusion **For ADAMTS14 rs4747096 genotype, the AA and AG genotypes significantly increased the risk of KOA. The recessive pattern of inheritance, older age, morbid obesity, and prolonged standing at work were the predictive risk factors for KOA. Further studies with larger sample size are encouraged to investigate the mechanism by which this genotype can affect the development of KOA.

## Introduction

Osteoarthritis (OA) is a degenerative disease with cartilage degradation, deterioration of the joint connective tissues, synovial inflammation, osteophyte formation, and bone remodeling [1–3]. It leads to chronic pain, absence of normal joint function, and eventually ends with impaired mobility and reduction of quality of life [1]. It is considered the fourth major cause of global Year Lived with Disability [4–6]. Moreover, patients with OA are at greater risk of sleep disturbances, depression, and all causes of death [7, 8]. Knee OA (KOA) is the most frequent arthropathies worldwide with a prevalence of 365 million of the adult population [9]. In Egypt, KOA prevalence is about 29.2 per 1000 which imposes a serious public health problem [10]. KOA is a multifactorial disease where risk factors at the joint level (abnormal loading of the joints, injury, mechanical stress, and malalignment) and person-level risk factors (age, gender, obesity, diet, occupation, metabolic syndromes as diabetes, and genetics) are all engaged in the development of the disease. It is more prevalent in developed than developing countries [7, 11].

OA arises from an inequality between synthesis and degeneration of extracellular matrix (ECM) which results in gradual cartilage loss. Thus, metalloproteinases (MMP) enzymes have been well-thought-out as the major hallmark of OA. A disintegrin and MMPs with thrombospondin motifs (ADAMTSs), have a crucial role in the degradation of cartilage of ECM [12]. ADAMTS14, a member of the ADAMTS family, is reported to be engaged in the formation process of collagen fiber II which is an essential component of joint cartilage. Abnormal metabolism of collagen fiber II leads to weakening of the joint cartilage so it is considered as an important contributor factor in the joint arthritis [13, 14]. ADAMTS14 gene rs4747096 is a non-synonymous coding single nucleotide polymorphism (SNP), sited on chromosome 10q22.1 [15]. This SNP can affect the amino acid sequence and the subsequent biological properties. Imbalance of ECM decomposition and synthesis could be due to abnormal expression of cytokines, protease, and other factors and this ultimately ends with cartilage loss [13]. Disorders or mutations in ADAMTS proteases have been linked to various diseases e.g., defective wound healing, defective angiogenesis, and arthritis [13, 16].

As the KOA continues to spread, knowledge of the risk factors that increase its severity is an important element. So, the present study aimed to examine the connection between SNPs of ADAMTS14 gene rs4747096 and KOA and to assess risk factors associated with KOA.

## Subjects and methods

A case–control study was carried out. It included 158 patients with KOA diagnosed according to American College of Rheumatology [17]. They were 114 females and 44 males, their ages ranged from 30 to 69 years. Patients were consecutively recruited from outpatient rheumatology clinic of Medical Services Unit at National Research Centre (NRC). KOA patients were excluded if they were suffered from severe liver or kidney dysfunction, severe cardiovascular diseases, malignant tumour, pregnant females, and patients with joint diseases other than OA (autoimmune diseases, rheumatoid arthritis, and gout). In addition, the controls were 120 individuals who had no signs or symptoms of osteoarthritis or joint disease, with comparable age and sex to patients with KOA. There wasn’t any parental relationship between the study subjects. The study was conducted in accordance with the Code of Ethics of the World Medical Association (Declaration of Helsinki). The study was approved as a part of a project by the Medical Research Ethical Committee of the NRC {registration number 19226}. Each participant provided an informed written consent after acknowledgement about the research. The study was conducted over a one year from July 2021- June 2022.

### Sample size and its basis

Assuming the power = 0.85 and α = 0.05, and by using PASS 11^th^ release [18], a sample of 126 in each of osteoarthritis and control groups is sufficient to find statistically significant difference between them regarding the frequency of GG expression in rs4747096 gene. Ma et al. [13] found that GG expression in rs4747096 gene is 21.4% among osteoarthritis group and 8.8% among control group. When the significant levels between osteoarthritis group and control group was achieved, so the study was terminated with 120 controls to save money.

### Tools

I. **Interview Questionnaires:**
  1. A face-to-face interview was conducted for both patients with KOA and controls to fulfil a questionnaire with special emphasis on demographic data, education, tobacco smoking, full medical history, family history of OA, physical activity, and working conditions related to OA.
    ⊠ All participants were assessed using Western Ontario and McMaster Universities Osteoarthritis Index (WOMAC) to evaluate pain, joint stiffness, and physical function. The WOMAC is a self-complete or interviewer-administered three-dimensional questionnaire, was used to assess health status and health outcomes in knee and/or hip OA. It included three sub-dimensions: pain (five items: using stairs, standing upright, sitting or lying, in bed, and during walking), joint stiffness (two items: after first waking and later in the day), and physical function (activities of daily living) (17 items: standing, bending, using stairs, walking, and rising from sitting) for the last 7 days. Scoring and psychometric properties: response options to WOMAC items were ‘None’ (score 0), ‘Mild’ (1), ‘Moderate’ (2), ‘Severe’ (3) and ‘Extreme’ (4). The pain score range from 0 to 20, stiffness score from 0 to 8, and physical function score from 0 to 68, with a total score of summing all the three sub-dimensions scores. Higher scores indicate worse pain, more joint stiffness and more physical function limitations. It was a Quantitative questionnaire and needs 10–15 min to be completed and 5–10 min to be scored [19]. WOMAC Validity: Construct validity when tested against other scales ranged from 0.68 to 0.75. WOMAC Reliability: Reliability estimates for pain, stiffness and physical function range from 0.73 to 0.96 [20].
II. **Clinical Assessment:**
  Thorough clinical examination with special emphasis on Knee, anthropometric assessment included weight, height for calculation of BMI, waist, hip were carried out. Blood pressure was assessed. In this clinical assessment, OA for some other joints weren’t ruled out.
III. **Laboratory analysis:**
  ⊠ Two ml venous blood samples were collected for all participants under complete aseptic conditions in sterile ethylenediaminetetraacetic acid (EDTA)-coated blood collection tubes for DNA extraction using DNA blood Mini Kit according to manufacturer’s instruction, then DNA concentration determination using Nanodrop 2000 Spectrophotometer then the genomic DNA was stored in the refrigerator at − 80°C until use.
  ADAMTS14/rs4747096 gene polymorphisms was detected by Real-Time polymerase chain reactions (PCR) using 7500 fast real time PCR system. PCR reactions was set up in 20 μl reaction volume including 50 ng DNA, 10 μl TaqMan genotyping Master Mix and 0.5 μl of 40X rs4747096 TaqMan genotyping assay in PCR strips. The PCR assay was carried out according to manufacturer’s instructions including one step of 10 min at 95 C followed by 40 cycles of DNA denaturation at 95C for 15s and annealing/extension at 60C for 1 min.

### Statistical analysis

Data entry and analysis were done using statistical program for social science (SPSS) version 26 for windows SPSS; Inc. (IL, USA). Frequency and percentages were computed for comparisons between qualitative variables. Continuous not normally distributed data was expressed as median and interquartile range. OR (odds ratio) was used to assess the degree of association. For comparing between two qualitative variables, chi square test was used and to compare between two medians, Mann–Whitney was used. For evaluating the association between median level of total WOMAC score and different ADAMTS14 gene rs4747096 genotypes among patients with KOA, Boxplot was done. The relation between KOA and ADAMTS14 gene rs4747096 polymorphism was assessed in four genetic patterns: the additive pattern (AA vs. GG and AG vs. GG), the allele pattern (mutation [A] vs. wild type [G]), the dominant pattern (AA-AG vs. GG), and the recessive pattern (AA vs. GG-AG). A Backward Wald multivariate logistic regression analysis for identifying the significant predicting variables for KOA among the studied participants who were genotyped was done according to different genetic patterns. Three different logistic models including the variables who were significant different in descriptive analysis were done and in each model one inheritance genetic pattern was considered as an independent variable. In model 1, the additive pattern genotype inheritance was included as a categorical independent variable. In model 2, the dominant pattern was included as an independent variable. While in model 3, the recessive pattern was included as an independent variable. *P* < 0.05 was considered statistically significant and *P* < 0.01 was considered statistically highly significant.

## Results

Among 158 patients with KOA and 120 controls, around half of the participants from each group were aged 55–64 years, about three quarters of them were females, and 40% finished the university stage of education, with no significant differences between patients and controls, *P* > 0.05.

Table (1) shows that the median total WOMAC score and levels of its domains; pain, stiffness, and physical function was highly significantly higher among patients compared to controls (*P* < 0.001).

**Table 1:** WOMAC test domains and total score among the studied participants

KOA WOMAC	Patients with KOA *N* = 158 (Median, IQR)	Controls *N* = 120 (Median, IQR)	*P* value
Pain	10.0 (6.3–13.0)	2.0 (0.0–4.0)	** < 0.001**
Stiffness	4.0 (3.0–5.0)	0.5 (0.0–2.3)	** < 0.001**
Physical function	36.0 (26.0–48.0)	5.0 (1.0–15.0)	** < 0.001**
Total WOMAC score	48.0 (39.0–63.0)	9.0 (3.0–21.0)	** < 0.001**

*IQR* Interquartile range, *P* value is highly significant if < 0.01

The ADAMTS14 gene rs4747096 allele frequencies revealed a Hardy–Weinberg Equilibrium, *P* > 0.05. The genotype distribution of the ADAMTS14 gene rs4747096 SNP among the patients with KOA was in the form of AA (73.5%), AG (25.7%), and GG (0.7%), whereas the genotype figure among the control group was AA (63.0%), AG (31.5%), and GG (5.6%). There was a statistically significant difference between the two groups as regards the frequency of different genotypes (AA, AG, and GG) and the frequency of different alleles (A and G), with more AA genotype and A allele among the patients (*P* = 0.038 and *P* = 0.025 respectively). Moreover, the A allele is a risk factor associated with KOA among male patients, it increased the chance of having KOA by significantly around five times, OR = 4.67 (Table 2).

**Table 2:** Genotype distribution and allelic frequency of ADAMTS14 gene rs4747096 polymorphism between patients with KOA and controls

**ADAMTS14 gene rs4747096**	**Patients with KOA** ***n*** **=136** **N (%)**	**Controls ** ***n*** **=108** **N (%)**	**OR (95%CI)**	***P*** **value**
**Genotype**				
AA	100 (73.5)	68 (63.0)	8.82 (1.04-74.95)*	0.046
AG	35 (25.7)	34 (31.5)	6.18 (0.71-54.04)	0.100
GG	1 (0.7)	6 (5.6)	**®**	
**Allele**				
A	235/272 (86.4)	170/216 (78.7)	1.72 (1.07-2.77)*	**0.026**
G	37/272 (13.6)	46/216 (21.3)	**®**	
**Genotype**^**a**^				
**AA **				
Male	32 (84.2)	22 (52.4)	NA	0.382
Female	68 (69.4)	46 (69.7)	2.96 (0.26-33.57)	
**AG**				
Male	6 (15.8)	16 (38.1)	NA	0.354
Female	29 (29.6)	18 (27.3)	3.22 (0.27-38.15)	
**GG**				
Male	0 (0.0)	4 (9.5)	**®**	
Female	1 (1.0)	2 (3.0)	**®**	
**Allele**				
**A**				
Male	70/76 (92.1)	60/84 (71.4)	4.67 (1.79-12.17)**	**0.002**
Female	165/196 (84.2)	110/132 (83.3)	1.06 (0.59-1.93)	0.837
**G**				
Male	6/76 (7.9)	24/84 (28.6)	**®**	
Female	31/196 (15.8)	22/132 (16.7)	**®**	

®Reference, *NA* Not applicable, *OR* Odds ratio

^*^P value is significant if < 0.05 and ^**^highly significant if < 0.01

^a^Each % of genotype is measured out of its total: total patients = 136 (38 males & 98 females) and total controls = 108 (42 males & 66 females)

Table 3 reveals the model of inheritance that had been analyzed in the two groups. It shows that the AA and AG genotypes significantly increased the risk of KOA, so the ADAMTS14/rs4747096 gene polymorphism is inherited by an autosomal dominance aspect (OR = 7.94, *P* < 0.05).

**Table 3:** Risk of KOA patients associated with ADAMTS14 gene rs4747096 A/G genotype according to different models of inheritance

ADAMTS14 gene rs4747096 model of inheritance	Patients with KOA *n* = 136 N (%)	Controls *n* = 108 N (%)	OR (95%CI)	*P* value
**A dominance**				**0.025**
AA-AG	135 (99.3)	102 (94.4)	7.94 (0.94–66.99)	
GG	1 (0.7)	6 (5.6)	®	
**A recessive**				0.077
AA	100 (73.5)	68 (63.0)	1.63 (0.95–2.82)	
GG-AG	36 (26.5)	40 (37.0)	**®**	

*P* value is significant if < 0.05

**Fig. 1:**
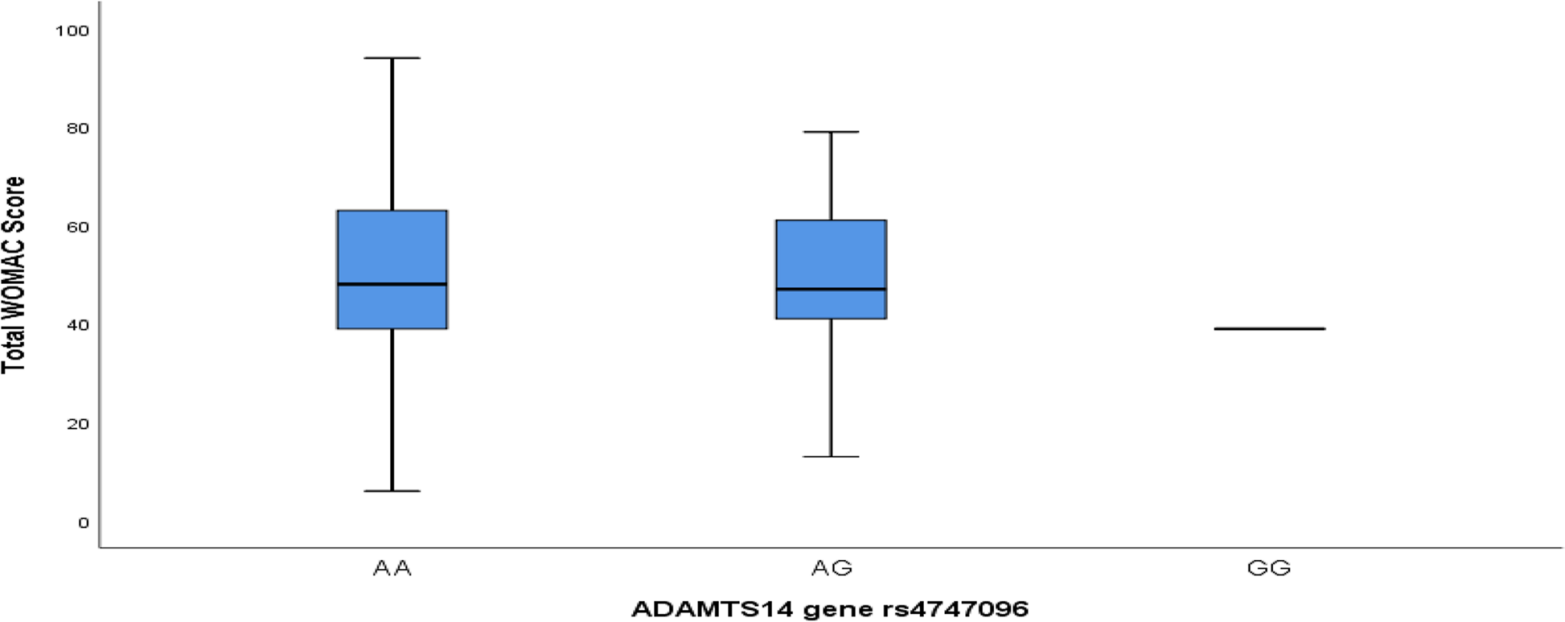
Boxplot of total WOMAC score and different ADAMTS14 gene rs4747096 genotypes among patients with KOA

Figure 1 displays that there was no significant association between median level of total WOMAC score and different ADAMTS14 gene rs4747096 genotypes among patients with KOA.

Table (4) shows that the risk of KOA among the studied participants who were genotyped was significantly higher among patients with older age, females, BMI ≥ 35, hypertension, and kidney disease compared to controls (OR = 3.3, 1.8, 4.8, 2.0, and 2.2 respectively, *P* < 0.01). Prolonged standing at work was significantly at higher risk of KOA among the studied patients compared to controls (OR = 2.6, *P* < 0.01). Lack of severe and moderate physical exercises were significantly increased the risk of KOA among patients compared to controls (OR = 3.0 and 2.1 respectively, *P* < 0.01).

**Table 4:** Risk factors associated with KOA among the studied participants who were genotyped

Variables	Patients with KOA *N* = 136 N(%)	Controls *N* = 108 N(%)	OR (95%CI)
**Age**			
• ≥ 50	112 (82.6)	63 (58.3)	(1.9–6.0)**
• < 50	24 (17.6)	45 (41.7)	®
**Age** (years, mean ± SD)	55.2 ± 7.1	51.6 ± 9.3	*p value: ***0.001**
**Sex**			
• Females	98 (72.1)	63 (58.3)	(1.1–3.1)*
• Males	38 (27.9)	45 (41.7)	®
**Education**			
• Secondary and less	47 (34.6)	47 (43.5)	(0.4–1.2)
• University and postgraduate stage	89 (65.4)	61 (56.5)	®
**BMI**^**a**^			
• ≥ 35	62 (45.6)	16 (14.8)	(2.6–9.0)**
• < 35	74 (54.4)	92 (85.2)	®
**BMI**^**a**^ (mean ± SD)	34.8 ± 6.8	30.6 ± 4.7	*p value:*** < 0.001**
**DM**^**b**^			
• Yes	48 (35.3)	30 (27.8)	(0.8–2.5)
• No	88 (64.7)	78 (72.2)	®
**Hypertension**			
• Yes	48 (35.3)	23 (21.3)	2.0 (1.1–3.6)*
• No	88 (64.7)	85 (78.7)	®
**High cholesterol**			
• Yes	52 (38.2)	33 (30.6)	(0.8–2.4)
• No	84 (61.8)	75 (69.4)	®
**Hormone replacement therapy**			
• Yes	4 (2.9)	1 (0.9)	(0.4–29.4)
• No	132 (97.1)	107 (99.1)	®
**Kidney disease**			
• Yes	39 (28.7)	17 (15.7)	(1.1–4.1)*
• No	97 (71.3)	91 (84.3)	®
**Working hours**			
• ≥ 7 h	88 (64.7)	74 (68.5)	0.8 (0.5–1.4)
• < 7 h	48 (35.3)	34 (31.5)	®
**Work type**			
• Manual	47 (34.6)	32 (29.6)	(0.7–2.2)
• Mental	89 (65.4)	76 (70.4)	®
**Different working positions**			
**1)Tashahhud mode at work**			
• Yes	25 (18.4)	18 (16.7)	(0.6–2.2)
• No	111 (81.6)	90 (83.3)	®
**2)Squatting position at work**			
• Yes	24 (17.6)	12 (11.1)	(0.8–3.6)
• No	112 (82.4)	96 (88.9)	®
**3)Bending at work**			
• Yes	69 (50.7)	53 (49.1)	(0.6–1.8)
• No	67 (49.3)	55 (50.9)	®
**4)Standing at work**			
• Yes	49 (36.0)	19 (17.6)	(1.4–4.8)**
• No	87 (*6*4.0)	89 (82.4)	®
**5)Climbing stairs at work**			
• Yes	81 (59.6)	53 (49.1)	1.5 (0.9–2.5)
• No	55 (40.4)	55 (50.9)	®
**6)Lifting heavy objects at work**			
• Yes	48 (35.3)	32 (29.6)	1.3 (0.9–2.2)
• No	88 (64.7)	76 (70.4)	®
**7)Riding car at work**			
• Yes	27 (19.9)	32 (29.6)	(0.3–1.1)
• No	109 (80.1)	76 (70.4)	®
**Physical exercise**			
**1)Severe physical exercise**			
• Inactive	116 (85.3)	71 (65.7)	(1.6–5.6)**
• Active	20 (14.7)	37 (34.3)	®
**2)Moderate physical exercise**			
• Inactive	54 (39.7)	26 (24.1)	(1.2–3.6)*
• Active	82 (60.3)	82 (75.9)	®
**3)Walking**			
• Inactive	16 (11.8)	13 (12.0)	1.0 (0.4–2.1)
• Active	120 (88.2)	95 (88.0)	®

®Reference

^a^*BMI* Body mass index

^b^*DM* Diabetes mellitus

*P value is significant if < 0.05 and **P value is highly significant if < 0.01

Table (5) demonstrates the results of Backward Wald multivariate logistic regression analysis for identifying the predicting variables affecting KOA among the studied groups who were genotyped according to different genetic patterns. By the three different logistic models, age ≥ 50, BMI ≥ 35, and prolonged standing at work were predictive factors for KOA (*P* < 0.01). In addition, only the A recessive pattern of inheritance presented as a predictive risk factor for KOA.

**Table 5:** Multivariate logistic regression analysis for identifying the predicting variables for KOA among the studied participants who were genotyped according to different genetic patterns

Variables	B	AOR	*P* value	95% CI for AOR	
				**Lower**	**Upper**
**Model 1**^**a**^					
Age ≥ 50	1.732	5.650	** < 0.001**	2.353	13.567
BMI ≥ 35	1880	6.554	** < 0.001**	2.512	17.097
Prolonged standing at work	0.837	2.308	**0.008**	1.246	4.276
Constant	-6.032	0.002	**0.000**		
**Model 2**^**b**^					
Age ≥ 50	1.705	5.504	** < 0.001**	2.276	13.312
BMI ≥ 35	1950	7.029	** < 0.001**	2.608	18.940
Prolonged standing at work	0.873	2.395	**0.007**	1.269	4.523
Constant	-6.032	0.002	**0.000**		
**Model 3**^**c**^					
Age ≥ 50	1.907	6.732	** < 0.001**	2.737	16.555
BMI ≥ 35	2.111	8.257	** < 0.001**	2.964	23.002
Prolonged standing at work	0.830	2.292	**0.010**	1.217	4.318
A recessive	0.921	2.511	**0.029**	1.101	5.730
Constant	-7.225	0.001	**0.000**		

*P* value is not-significant if > 0.05, significant if < 0.05 and highly significant if < 0.01

*AOR* Adjusted odds ratio, *CI* Confidence interval

^a^Variables entered in model 1 were additive pattern of ADAMTS14/rs4747096 gene polymorphisms, age, gender, BMI, hypertension, kidney disease, standing at work, and physical exercise

^b^Variables entered in model 2 were dominance pattern of inheritance of ADAMTS14/rs4747096 gene polymorphisms, age, gender, BMI, hypertension, kidney disease, standing at work, and physical exercise

^c^Variables entered in model 3 were recessive pattern of inheritance of ADAMTS14/rs4747096 gene polymorphisms, age, gender, BMI, hypertension, kidney disease, standing at work, and physical exercise

## Discussion

KOA is a musculoskeletal disease characterized by progressive cartilage loss, usually presenting by chronic pain, limitation of movement, and disability [21]. In the current work, the median WOMAC score was 48 with IQR: 39–63, so severe knee affection was remarkable among most of OA patients as the total WOMAC score (a disease-specific grading scale) is 96. As expected, this patients’ WOMAC score was significantly higher compared to controls (with median 9 and IQR: 3–21), *P* < 0.001. Furthermore, the three subscales of WOMAC index score; pain, stiffness, and physical activities showed significantly higher values among KOA patients compared to controls. Similar results were reported by other studies [22–25].

In agreement with previous studies [26–29], this current work found that older age ≥ 50 years was associated with higher risk of KOA with an AOR more than 5 times compared to younger age. This could be interpreted by the degenerative changes occur in the cartilage, the osteophytes development, and the decreased muscle mass that occur with old age resulting in joint destruction. Moreover, increased matrix MMP production and proinflammatory cytokines associated with the aging process decrease matrix synthesis, increase susceptibility to cell death and trigger joint degradation. These conditions may be end by joint cartilage damage [29].

The current study revealed that females were at higher risk of the KOA with OR = 1.8 (95%CI: 1.1–3.1), *P* < 0.05 and this result was in adherence to prior studies [30, 31]. Furthermore, the loss of the protective effect of estrogen hormone on articular cartilage could be the cause of rapid progression of KOA among post-menopausal women. These annotations, together with anatomical variations between men and women, that represented by thinner cartilage and greater valgus knee angle, may explain the higher rate of articular cartilage loss and the greater KOA severity in women [32, 33]. Moreover, estrogen contributes in nerve growth factor (NGF) regulation by decreasing NGF expression in chondrocytes, which is essential for the development of OA pain. Accordingly, estrogen plays a role in decreasing OA pain, which explain the postmenopausal severity of KOA symptoms. On the other hand, androgens are the precursors for estrogen. Similar to estrogen, androgens take part in cartilage protection. Androgen levels are high in men and low in women, this could explain why men have lower risk of OA [34].

The present study findings show evidence that obesity was one of the risk factors for KOA as demarcated by high BMI ≥ 35 that significantly increased the risk of the disease with an AOR more than 6 times risk compared to lower BMI. This was in accordance with previous studies stating that obesity was one of the major risk factors for KOA [35–39], and that every 5-unit rise in BMI results in increasing the risk of KOA by 35% [40]. In addition, Visser et al. [41] had found an association between high mass/skeletal muscle ratio and KOA. Moreover, in agreement with this study, Szilagyi et al. [42] stated that obesity and female gender represent vital risk factors for KOA. This association can be attributed to the metabolic, biological, and immunological sequels of the obesity [36]. A study done by van der Esch et al. [43] found that obesity was one of the five different OA phenotypes. While a systematic review conducted by Dell’Isola et al. [44] reviewed 24 studies concluded that there were five different OA phenotypes and one of them was metabolic syndrome. They suggested that patients with OA had higher prevalence of metabolic factors as obesity, diabetes, hypertension, and dyslipidemia. In addition, the greater body weight increases the mechanical weight-bearing pressure on the knee joint and increases the tumor necrosing factor α (TNFα) and interleukin-1β (IL-1β) levels that activate the ruin of the ECM cartilage, thereby increasing the probability of KOA. Furthermore, the low-grade systemic inflammation caused by some molecules such as interleukin-6 (IL-6), IL-1β, TNFα, leptin, and adiponectin may play a role in obesity association with KOA [40, 45, 46].

Hypertension and kidney disease significantly increased the risk of KOA by around two times as shown by current study, OR (95%CI) were 2.0 (1.1–3.6) and 2.2 (1.1–4.1) respectively, *P* < 0.05. These findings were consistent with a study done by Ren et al. [28]. Additionally, the pooled results of two meta-analyses performed by Zhang et al. and Lo et al. [47, 48] showed that hypertension was significantly associated with increase the occurrence of KOA. Moreover, according to the results of a study done on Korean population, there was a significantly higher prevalence of KOA among hypertensive patients [49]. In addition, Puenpatom and Victor [50] detected higher prevalence of hypertension among US patients with OA than in those without OA. Hypertension could be an isolated risk factor for KOA. However, because hypertension has been known to be associated with many overlapping factors, it is still debatable that KOA is associated with hypertension. Common risk factors, such as obesity, aging, and chronic inflammation have been involved in the probable mechanisms of the relation between hypertension and KOA, in addition to the vital role played by the proinflammatory cytokine interleukin-6 in both diseases [51]. Regarding the reason behind kidney disease association with KOA, it could be the medication used especially nonsteroidal anti-inflammatory drug (NSAID) treatment to alleviate the associated pain in addition to the presence of chronic inflammation in both diseases [28].

In the current study, prolonged standing and severe physical activity were reported as important risk factors for KOA in the univariate analysis [OR (95%CI)= 2.6 (1.4-4.8) and OR (95%CI)= 3.0 (1.6-5.6) respectively], *P* < 0.01, while in the three different logistic models, prolonged standing was a predictive value for KOA (AOR= around 2.0), *P* < 0.05. These findings are highly consistent with previous systematic reviews which stated that physically demanding occupations that requires prolonged standing as well as strenuous efforts were all associated with a higher risk of KOA [52]. Lementowski and Zelicof [36] stated that severe mechanical forces exerted on the joints are a considerable cause of OA.

Besides the above investigated risk factors, the ADAMTS14 rs4747096 genotype was examined among KOA patients and controls and it was found a statistically significant difference between the two groups. The frequency of ADAMTS14 gene rs4747096 genotypes among patients with KOA was 73.5% for AA, 25.7% for AG, and 0.7% for GG compared to controls 963%, 31.3% and 5.6% respectively and the frequency of alleles among patients was 86.4% for A and 78.7% for G compared to controls (78.7% and 21.3% respectively, *P* < 0.05. This was in line with the findings of a meta-analysis performed by Li et al. [53]. Most previous studies of different populations have found a significant association between the ADAMTS14 rs4747096 genotype and the susceptibility to OA regarding the genotype distribution [13, 54–57] and the allele frequency [54, 56] indicating this genotype as a high-risk factor for OA. In agreement with the current results, Mostafa et al. [56] investigated the ADAMTS14 rs4747096 genotype among Egyptian population (42 KOA and 31 controls without OA) and showed that AA genotype frequency was significantly higher among patients compared to the control group and the A allele was significantly more frequent among KOA patients. In accordance, a Turkish study conducted by Haberal et al. [58] on 300 KOA patients, AA were the most common genotype. Furthermore, in a study conducted by Poonpet et al. [54] among 108 Thai patients with severe KOA and 119 controls without OA, there was a significant association between the AA genotype and KOA and between A allele and KOA among female patients. However, they did not find any association between the rs4747096 polymorphism and KOA among males.

In contrast to the present results, Ma et al. [13] examined the ADAMTS14 rs4747096 genotype among 346 Chinese Han KOA patients and 480 controls found that the G allele was significantly higher among KOA patients than in the control group and observed significant differences in the genotype frequency. Also, they showed that the GG genotype and the GG/AG combination were more frequent among patients than in controls without OA. Furthermore, Rodriguez-Lopez et al. [55] found that the GG genotype was significantly associated with KOA among European Caucasians patients and the difference was clear between patients and controls with predominance in women. Additionally, in the women patients, they observed some indication that G allele has dominant effect.

The univariate genetic association analysis under different inheritance models among the studied group demonstrated an inheritance of ADAMTS14 rs4747096 polymorphism with an autosomal dominance aspect model, since the AA and AG genotypes significantly increased the risk of KOA, OR = 7.9 (95%CI: 0.97–70.0), *P* < 0.05 compared to recessive model, OR = 1.6 (95%CI: 0.95–2.8), *P* > 0.05. In consistent with the current findings, Poonpet et al. [54] found association of ADAMTS14 rs4747096 polymorphism with female KOA patients and showed that the inheritance model of ADAMTS14 rs4747096 gene polymorphism was of an autosomal dominance aspect and the AA and AG genotypes significantly increased risk of the disease. These results differ from the inheritance model findings of Ma et al. [13] who concluded that the GG genotype and the GG/AG combination were more common among KOA patients than controls and elevated the risk of KOA in the dominant model. Additionally, Rodriguez-Lopez and colleagues [55] indicated a dominant aspect of the G allele in the women KOA patients. Meanwhile, another Turkish study noticed that there was no statistically significant relationship between severe KOA and the ADAMTS14 rs4747096 polymorphisms among 300 KOA patients [58]. There was no significant association between the severity of KOA (median level of total WOMAC score) and different ADAMTS14 gene rs4747096 genotypes among patients with KOA. Using the multivariate logistic analysis revealed that only the A recessive pattern of inheritance was a significant predictive risk factor for KOA. The contradictory results about the relation between KOA and ADAMTS14 gene rs4747096 SNP in different studies may be due to different genetic distributions between different ethnic populations and different polymorphisms of the same gene affecting the development of KOA. In addition, these conflicting results could be attributed to the sample size, age and sex of the participants, and the severity of the disease. Moreover, geographical factors and environmental conditions such as lifestyle, physical activity, and diet can cause variations in gene-to-gene interactions [58].

## Conclusion

This study found that there was a significant association between the ADAMTS14 rs4747096 genotype and KOA was detected regarding the genotype distribution and the allele frequency, as the AA genotype and A allele were more frequent among the KOA patients. In addition, the A allele was a risk factor associated with KOA among male patients. The AA and AG genotypes significantly increased the risk of KOA. Using multivariate logistic analysis, the A recessive pattern of inheritance, older age ≥ 50 years, BMI ≥ 35, and long standing at work were the predictive risk factors for KOA. As KOA reduced the patients’ quality of life and their physical and psychological conditions, further studies are encouraged to investigate the mechanism by which this genotype can affect the development of KOA with larger samples among the Egyptian population. This might be a basis for KOA prevention and treatment in the future.

### There are some limitations in the current study

The study only assessed the association between KOA and SNPs of ADAMTS14 gene rs4747096, but not other gene types and this could lead to unsuitable decision about the genetic effect on KOA risk. The results cannot be representative or generalized because of the small sample size and the sample was taken from a single center.

## Data Availability

The datasets used and/or analyzed during the current study are available from the corresponding author on reasonable request.

## Abbreviations

KOA: Knee osteoarthritis
OA: Osteoarthritis
KOA: Knee OA
ECM: Extracellular matrix
MMP: Metalloproteinases
ADAMTSs: A disintegrin and MMPs with thrombospondin motifs
SNP: Single nucleotide polymorphism
NRC: National Research Centre
BMI: Body mass index
WOMAC: Western Ontario and McMaster Universities Osteoarthritis Index
PCR: Polymerase chain reactions
SPSS: Statistical program for social science
OR: Odds ratio
AOR: Adjusted odds ratio
CI: Confidence interval
IQR: Interquartile range
NGF: Nerve growth factor
TNFα: Tumor necrosing factor α
IL: Interleukin
